# Endoscopic endonasal intradural pituitary transposition for resecting retroinfundibular lesions: technique notes and a single institute experience

**DOI:** 10.3389/fendo.2025.1547980

**Published:** 2025-09-29

**Authors:** Daibo Ke, Shaocheng Yang, Yifeng Lin, Hao Liu, Wei Chen, Tao Lv, Xiang Yue, Ling Xu, Shunwu Xiao

**Affiliations:** ^1^ Department of Neurosurgery, The Affiliated Hospital of Zunyi Medical University, Zunyi, China; ^2^ Graduate School, Zunyi Medical University, Zunyi, China

**Keywords:** retroinfundibular area, pituitary gland transposition, Intradural, dorsectomy, posterior clinoidectomy

## Abstract

**Background:**

The endoscopic endonasal approach (EEA) is the mainstay of resection for lesions in the retroinfundibular area and the prepontine and interpeduncular cisterns. Owing to the anatomical barrier of structures such as the pituitary gland (PG)/pituitary stalk (PS), dorsum sellae (DS) and posterior clinoid process (PCPs), sufficient tumour resection often requires displacement of the pituitary gland and varying degrees of bony resection.

**Methods:**

We retrospectively studied the clinical data of 23 patients, from June 2016 to February 2023,who underwent endoscopic endonasal intradural pituitary gland transposition (PGT) as well as dorsectomy and posterior clinoidectomy for the treatment of lesions involving the retroinfundibular area, prepontine cistern and interpeduncular cisterns. Outcomes, including postoperative complications and the extent of tumour resection (EOR), were evaluated.

**Results:**

Among the 23 patients with tumours, 16 had craniopharyngiomas, 3 had germ cell tumours, 2 had epidermoid cysts, and 2 had gliomas. Fifteen patients underwent unilateral PGT and ipsilateral dorsectomy, and 8 patients underwent ipsilateral posterior clinoidectomy. Ten patients with visual impairment improved, and none of the patients experienced cranial nerve palsy postoperatively. Fourteen patients developed hypopituitarism, and 8 patients experienced diabetes insipidus (DI) postoperatively, 6 and 4 of theses patients recovered after 2–4 weeks of replacement therapy. Twelve patients with intraoperative high-flow CSF leakage underwent an average of 7 days of early postoperative lumbar drain (LD). Among them, 4 patients developed an infection, which was cured by 10 days of antibiotic treatment combined with LD. None of the patients experienced constant CSF leakage at the discharge. Gross total resection (GTR) was achieved in 19 tumour patients, and near-total resection (NTR) was achieved in 4 patients. The average follow-up period was 26 months, and magnetic resonance imaging (MRI) revealed no tumour recurrence in 22 patients.

**Conclusion:**

Tumours of the retroinfundibular area, prepontine and interpeduncular cisterns can be safely removed via the PGT technique. The intradural PGT technique combined with flexible dorsectomy and posterior clinoidectomy has obvious advantages, including less intraoperative bleeding, more effective pituitary transposition, and good preservation of pituitary function. Owing to the complexity of these regions, this technique should be performed by experienced endoneurosurgeons.

## 1 Introduction

The retroinfundibular area, prepontine and interpeduncular cisterns are difficult to access during surgery. In the past, various cranial base approaches have been used for the resection of lesions in these areas and in different groups of patients with neurovascular injuries (1–3). With the development and modification of neuroendoscopic techniques, the EEA has become the optimal choice for the resection of lesions in these regions (4–8). As the pituitary gland(PG)/pituitary stalk (PS), dorsum sellae (DS), and posterior clinoid process (PCPs) represent the natural anterior barriers to these regions when the endoscopic endonasal approach (EEA) is undertaken, pituitary transposition with reasonable DS and PCP resection is needed to ensure an adequate working space and satisfactory tumour resection.

Pituitary gland transposition (PGT) can be divided into extradural, interdural, and intradural types according to how the dural layers and pituitary ligaments are dissected (9–12). Each PGT technique has advantages and disadvantages. In this study, we described the treatment of intradural PGTs with reasonable DS and PCP resection through the EEA in 23 patients. We believe that the intradural PGT technique combined with reasonable bone removal has obvious advantages, including less intraoperative bleeding, more effective pituitary transposition, and good preservation of pituitary function.

## 2 Materials and methods

The data of twenty-three patients who underwent surgical treatment between June 2016 and February 2023 were retrospectively analysed. All these patients underwent endoscopic endonasal PGT in addition to dorsectomy and posterior clinoidectomy. This study was approved by the Institutional Review Board of the Affiliated Hospital of Zunyi Medical University, and all the patients signed informed consent forms, which permitted the publication of their data.

### 2.1 Patient characteristics

There were 10 males and 13 females, with an average age of 37.8 ± 15.5 years. The clinical data included clinical symptoms, preoperative visual field examination findings and pituitary function, postoperative visual outcomes and pituitary function, surgical complications, pathological diagnoses and follow-up results. All these data were collected and are listed in **Table 1**.

**Table 1:** Clinical characteristics and outcomes of the 23 patients with retroinfundibular lesions.

No.	A/G	Pre-op symptoms	Pre-op ED	Bone drilling	Tumor type	Pos-op Complications	EOR	LD (Yes/No)	Visual change	Follow-up /month
1	17/M	Headache	HPT	DS	Craniopharyngioma	DI	GTR	Yes	None	46
2	23/	DI	HPA	DS	Craniopharyngioma	HPA	GTR	Yes	None	43
3	10	Headache+VI	HPT	DS	Craniopharyngioma	HPT	GTR	No	Improved	42
4	37	Headache	PHP	DS+PCP	Craniopharyngioma	PHP+ DI+infection	GTR	Yes	None	40
5	49	Headache+VI	HPA	DS+PCP	Craniopharyngioma	PHP+ DI	GTR	Yes	Improved	38
6	40	Headache	HPT	DS	Craniopharyngioma	None	GTR	No	None	37
7	19	Headache+VI	HPA	DS+PCP	Craniopharyngioma	PHP+DI+ infection	GTR	Yes	Improved	36
8	48	DI	HPT	DS	Craniopharyngioma	DI+HPA	GTR	No	None	35
9	60	Headache+VI	PHP	DS+PCP	Craniopharyngioma	HPA	GTR	Yes	Improved	35
9	60	Headache+VI	PHP	DS+PCP	Craniopharyngioma	HPA	GTR	Yes	Improved	35
10	57	DI	None	DS	Craniopharyngioma	None	GTR	No	None	28
12	44	Headache	None	DS	Germ-cell tumor	None	GTR	No	None	25
13	46	Headache	HPA	DS	Craniopharyngioma	HPA+DI	GTR	Yes	None	24
14	64	None	None	DS	Epidermoid cysts	None	GTR	No	None	22
15	32	VI	None	DS	Glioma	None	NTR	No	Improved	20
16	70	Headache+VI	HPT	DS+PCP	Craniopharyngioma	DI+HPA+ infection	GTR	Yes	Improved	19
17	62	None	HPA	DS	Craniopharyngioma	HPA	GTR	No	None	17
18	34	VI	HPT	DS	Germ-cell tumor	None	NTR	No	Improved	17
19	50	Headache+VI	PHP	DS+PCP	Craniopharyngioma	PHP+ DI	GTR	Yes	Improved	15
20	71	Headache	None	DS	Germ-cell tumor	None	NTR	No	None	14
21	27	None	None	DS	Epidermoid cysts	None	GTR	No	None	10
22	57	Headache+VI	None	DS+PCP	Glioma	HPA	NTR	Yes	Improved	8
23	39	Headache	HPA	DS	Craniopharyngioma	HPT	GTR	Yes	None	6

A, age; G, gender; Pre-op, pre-operation; DI, diabetes insipidus; VI, vision impairment; ED, endocrine function; HPT, hypothyroidism; HPA, hypoadrenalism; PHP, panhypopituitarism; DS, dorsum sellae; PCP, posterior clinoid process; Pos-op, post-operation; EOR, extent of resection; GTR, gross total resection; NTR, near-total resection; LD, lumber drain.

### 2.2 Imaging evaluation

Preoperative MRI was performed to evaluate the size, shape and relationship with the PG/PS, optic chiasma (OC) and anterior communicating artery (AComA). A thin-layer three-dimensional computed tomography (CT) scan was used to examine the skull base, including the DS and PCPs. The relationship between the lesion and these bony structures was preliminarily evaluated. Head CT angiography (CTA) was used to examine whether there was an unruptured aneurysm or if the internal carotid artery (ICA) and its major branches had an abnormal course. The first follow-up MRI was performed within 3 days after surgery to evaluate the EOR. Thereafter, head MRI was performed at 3, 6, and 12 months after discharge.

### 2.3 Inclusion criteria for the surgical approach

PGT is based mainly on preoperative imaging features and clinical experience. In this study, all 23 patients who underwent PGT presented the following features: 1. Preoperative MR images revealed a normal morphology of the pituitary gland, indicating that the tumour did not originate from the pituitary gland. 2. All lesions were located behind the PG/PS, with the main body located at the midline or slightly deviated to one side. 3. The inferior portion of the tumour is located behind the dorsum sellae or occupies the prepontine and/or interpeduncular cisterns.

## 3 Surgical technique

### 3.1 Initial stage

The surgical position, nasal disinfection, nasal mucosa blood vessel contraction and pedicled vascularized nasoseptal flap (PVNF) have been described in previous articles (13, 14). All the septums of the sphenoidal sinus were carefully removed to expose the anatomical landmarks. To avoid injuring the cavernous ICA, we used a 3-mm diamond burr in the bone drilling process whenever possible. We usually make an en bloc bone flap if the sellar prominence is intact, which can be subsequently used for skull base reconstruction.

### 3.2 Relevant anatomy

**Figure 1:**
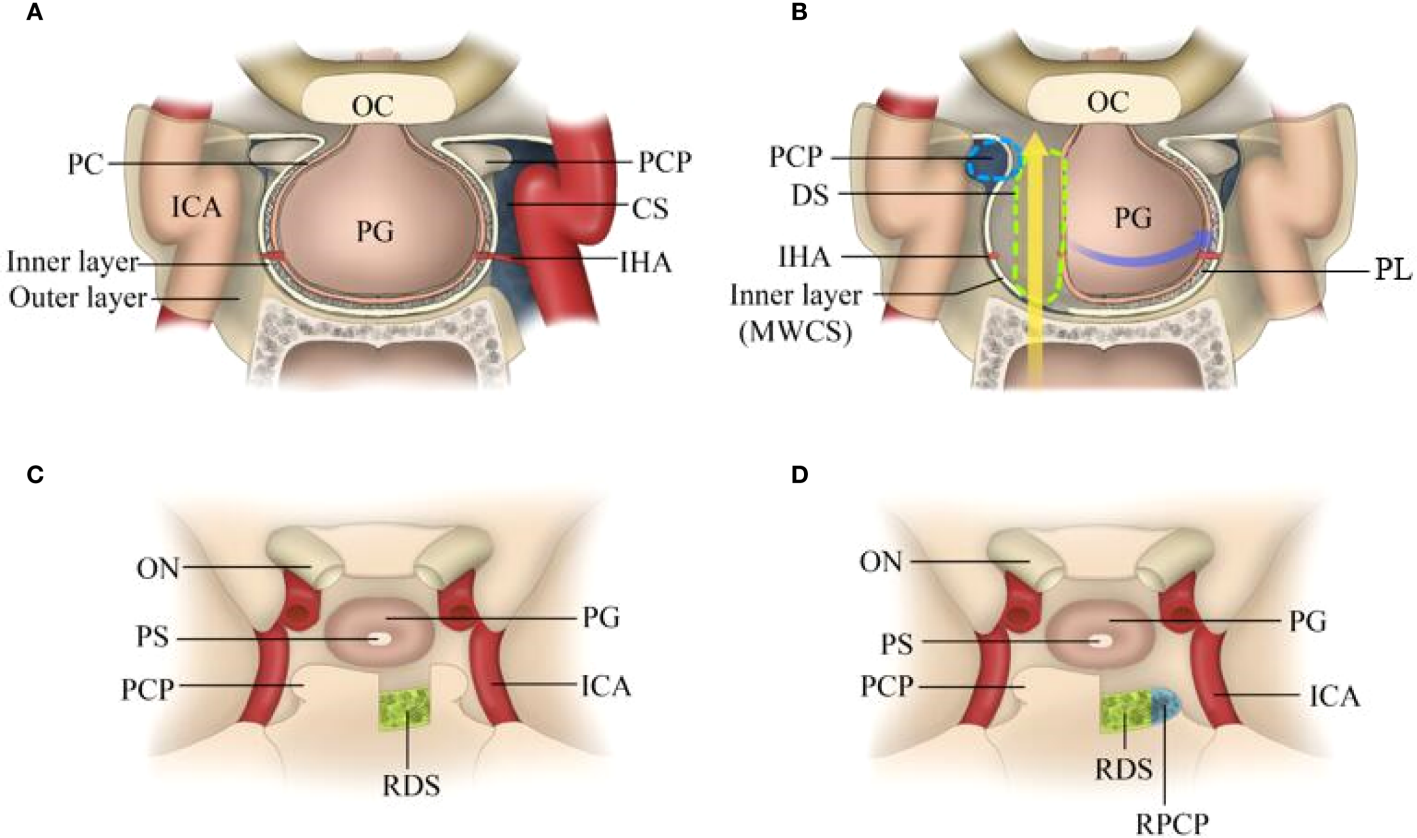
Schematic of intradural pituitary transposition and bone removal. **(A)** Illustration showing the membrane structure of the PG (three layers in the anterior-posterior-superior-inferior walls and two layers in both lateral walls). **(B)** Illustration showing the corridor between the pituitary capsule and the MWCS. After the dura was incised and the “pituitary ligaments” were split, the ipsilateral IHA was cut, and the PG and PS were displaced and rotated anteriorly and contralaterally to provide necessary access for the resection of the DS and PCPs. **(C)** Illustration showing that the DS was resected with PCPs preserved. **(D)** Illustration showing that the DS and ipsilateral PCP were resected. OC, optic chiasma; PG, pituitary gland; PCP, posterior clinoid process; CS, cavernous sinus; IHA, inferior hypophyseal arteries; MWCS, medial wall of the cavernous sinus; PL, pituitary ligament; ON, optic nerve; PS, pituitary stalk; RDS, right dorsum sellae; RPCP, right posterior clinoid process.

A better understanding of the membrane structure of the sellar/parasellar region is highly important for performing PGT **Figure 1**. The dura mater that surrounds the PG is composed of 2 layers—the periosteal (outer) layer and the meningeal (inner) layer. Both dural layers split and separate from each other at the lateral limit of the sella. The periosteal layer runs laterally to constitute the anterior wall of the cavernous sinus (AWCS), whereas the meningeal layer remains attached to the PG to form the medial wall (or sellar part) of the CS (MWCS). Therefore, both the AWCS and the MWCS are single layered with distinct origins, and the MWCS separates the PG from the CS. The third membrane surrounding the PG is the pituitary capsule, which is a thin layer of connective tissue that immediately covers and protects the PG. The pituitary capsule is loosely connected to the meningeal layer of the dura mater by numerous fibrous projections named the pituitary ligaments. After these ligaments and inferior hypophyseal arteries (IHAs) were systematically transected, the PG was completely free and transposed circumferentially.

### 3.3 Dural opening and tumour exposure

**Figure 2:**
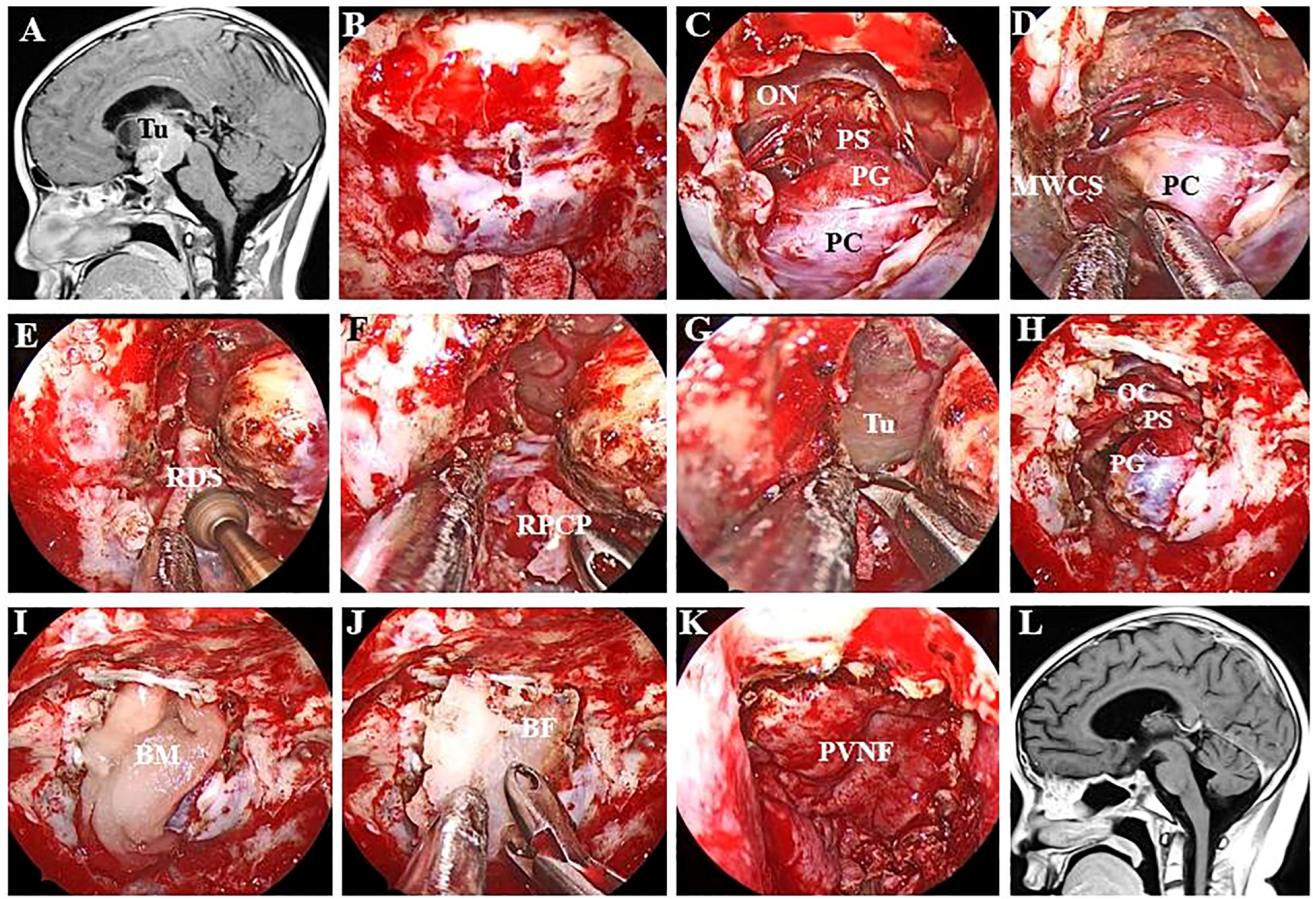
Endoscopic endonasal view of the procedure and nuances of intradural PGT. **(A)** Preoperative MR image showing a large craniopharyngioma in the retroinfundibular area. **(B)** A longitudinal dural incision was made at the site of the sellar tubercle. **(C)** The dura mater was extended upwards and downwards without injuring the pituitary capsule. **(D)** The pituitary ligament between the MWCS and the pituitary capsule was excised, after which the PG was displaced laterally. **(E)** The DS was resected via high-speed drilling, and the clival dura was exposed. **(F)** The right PCS was removed to provide more space for manipulation inside the interpeduncular cistern. **(G)** The clival dura was cut downwards to expose the tumour more effectively. **(H)** The structure of the PG and optic chiasma was intact after complete tumour resection. **(I)** An absorbable artificial biomembrane was placed as the first step. **(J)** An *in situ* bone flap was placed on the biomembrane for complete osseous reconstruction. **(K)** A vascularized pedicled nasoseptal flap was used to repair the defect. **(L)** MRI revealed no residual tumour or recurrence. Tu, tumour; ON, optic nerve; PS, pituitary stalk; PG, pituitary gland; PC, pituitary capsule; MWCS, medial wall of the cavernous sinus; RDS, right dorsum sellae; RPCP, right posterior clinoid process; BM, biomembrane; BF, bone flap; PVNF, pedicled vascularized nasoseptal flap.

Opening the dura is the key to this procedure **Figure 2**. The authors favour intradural transposition, cutting the pituitary ligaments and IHA in the space of the MWCS and pituitary capsule, avoiding opening the CS laterally and violating the tight pituitary capsule medially. One-sided PGT (hemitransposition) was performed on the ipsilateral side of the tumour, and a contralateral approach was utilized only for the treatment of tumours located behind the dorsum sellae and ICAs.

At the beginning of the procedure, the dura overlying the tuberculum was vertically incised, and then the superior intercavernous sinus was ligated and transected. The dural opening was subsequently extended superiorly and inferiorly to communicate with the suprasellar and sellar regions. After sharp dissection of the suprasellar arachnoid and early exposure of the PS, superior hypophyseal artery (SHA) and optic chiasma (OC), the dural opening is further extended laterally and inferiorly until the inferior intercavernous sinus is coagulated and cut to debond the anterior sellar dura bands early. These steps result in a wide intradural corridor to split the pituitary ligaments and cut off the ipsilateral IHA, after which the PG/PS can be displaced and rotated anteriorly and contralaterally without any dural attachment.

**Figure 3:**
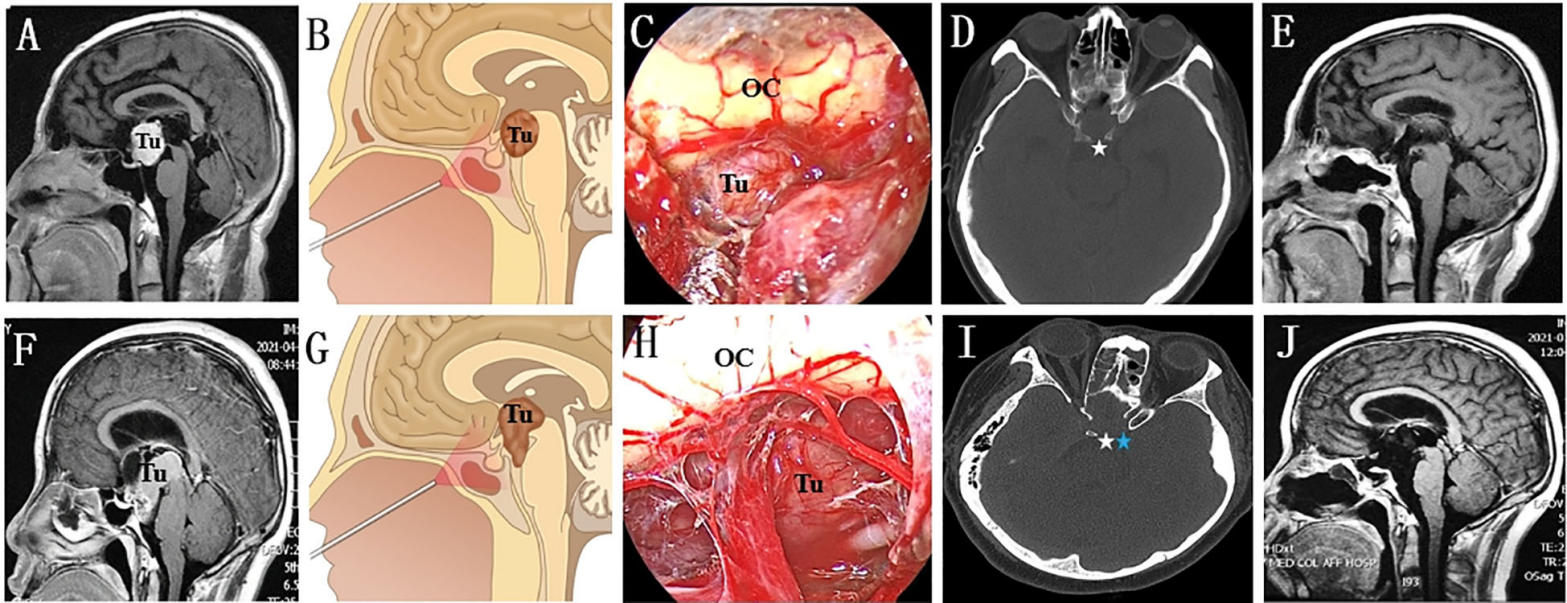
Endoscopic endonasal intradural pituitary transposition for resecting retroinfundibular lesions. **(A, B)** The tumour is located retroinfundibularly and extends inferiorly into the prepontine areas. **(C)** The infrachiasmatic space was narrow, and the tumour was blocked by the PG and DS. **(D)** The DS was individually drilled out (white arrow) and confirmed by a postoperative thin-layer CT scan. **(E)** Follow-up MR image showing that the tumour was radically resected. **(F, G)** This retroinfundibular tumour was very large and occupied both the prepontine and interpeduncular cisterns. **(H)** Except for the DS, the surgical corridor was effectively enlarged by removing the left PCP. **(I)** The left DS (white arrow) and PCP (blue arrow) disappeared on the postoperative thin-layer CT scan. **(J)** Follow-up MR image showing no residual tumour or occurrence. Tu, tumour; OC, optic chiasma.

The decision to perform dorsectomy and/or posterior clinoidectomy is contingent on the involvement of the tumour. If the tumour (**Figure 3**) extends downwards and occupies the prepontine areas, a second sellar floor dura incision, unilateral dorsectomy and posterior fossa dura incision are needed following PGT. For lesions with lateral extension or involving the interpeduncular cistern, the ipsilateral side of the PCP, which is detached from the upper clivus when the dorsum sellae is drilled out, must be carefully removed to create additional working space in the interpeduncular cistern.

### 3.4 Intradural tumour resection

**Figure 4:**
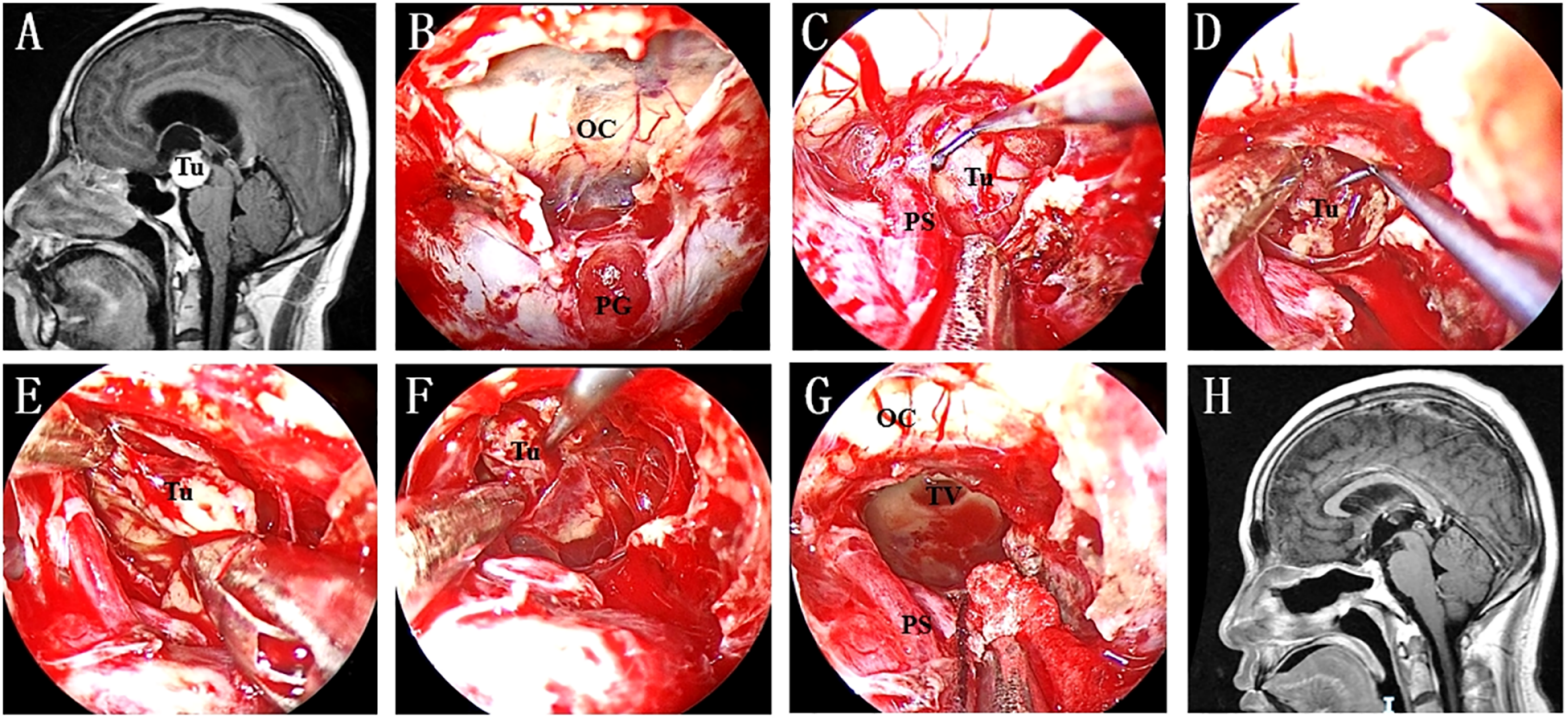
**(A)** Preoperative MR image showing a lesion in the retroinfundibular area. **(B)** A “Y”-shaped dural incision was made to expose the PG and optic chiasma. **(C)** The lesion was exposed after intradural PGT and clival dura opening. **(D)** Intratumoral debulking was performed as the first step to ensure extensive tumour resection. **(E)** The medial interface was identified after enough tumour debulking. **(F)** Inferior extracapsular dissection was continued to achieve radical tumour resection. **(G)** The TV was opened after the tumour was completely resected. **(H)** No residual tumour or recurrence was found on postoperative MRI. Tu, tumour; OC, optic chiasma; PG, pituitary gland; PS, pituitary stalk; TV, third ventricle.

Although the specific techniques vary, endoscopic resection of retroinfundibular lesions is considered a microsurgical operation **Figure 4**. Ring curettes and suction are the most commonly used tools for intratumoral debulking. Following this first step, extracapsular dissection was carried out at the interface, if it could be identified, between the tumour capsule and arachnoid planes. Under direct visualization of neurovascular structures, including the PG/PS, SHA and OC, the arachnoid bands covering the tumour capsule are sharply and carefully separated, avoiding injuring the small neurovascular perforators. We strongly recommend discontinuing the procedure if these arachnoid bands cannot be sharply resected. For lesions without a macroscopic encapsule, negative margins can be ensured based on tumour texture and surgical experience.

### 3.5 Skull base reconstruction

The mainstream technique for skull base reconstruction is multilayered closure, which is properly adjusted based on the defect size and the volume and location of cerebrospinal fluid (CSF) leakage during the operation (**Figure 2**). Classic three-layered repair of small and larger defects with low-flow CSF leaks was described in our previous study (13, 14). For lesions with large dura defects and high-flow CSF leaks, which are encountered in most of these cases, in addition to a subdural inlay graft as the first defence to counter CSF pressure and the previously reserved PVNF as the third layer of reinforcement, an onlay of osseous support (*in situ* bone flap, nasal septum, or vomer) is used as the second layer to provide rigid buttress. Furthermore, patients with high-flow CSF leakage, especially those with third-ventricle opening, underwent LD in the early period.

## 4 Results

The cohort included 16 craniopharyngiomas, 3 germ cell tumours, 2 epidermoid cysts, and 2 gliomas. All 23 patients underwent intradural PGT. Fifteen patients received unilateral PGT and ipsilateral dorsectomy, while 8 patients underwent a second ipsilateral posterior clinoidectomy. No patients developed postoperative consciousness impairment or neurological morbidity. Ten patients with preoperative vision impairment showed remarkable improvement, and the remaining 13 patients with normal baseline visual acuity maintained their visual function postoperatively.

Fourteen patients developed postoperative hypopituitarism: 8 presented with hypoadrenalism, 4 with panhypopituitarism, and 2 with hypothyroidism. Six patients recovered within 2–4 weeks after hormone replacement therapy. Additionally, 8 patients developed postoperative diabetes insipidus (DI); 4 recovered after 2 weeks of 1-deamino-8-d-arginine vasopressin (DDAVP) treatment. The remaining 8 patients with persistent hypopituitarism and 4 with unrelieved DI required ongoing postoperative administration of hydrocortisone acetate and DDAVP, respectively.

Twelve patients with intraoperative high-flow CSF leakage underwent early postoperative lumbar drain (LD) for an average of 7 days. Among them, 4 developed infections, which were resolved with 10 days of antibiotic therapy combined with LD. No patients had persistent CSF leakage at discharge. Gross-total resection (GTR) was achieved in 19 patients, and near-total resection (NTR, >95% tumour volume removal) in the rest. After a mean follow-up of 26 months, 2 patients with germ cell tumours experienced recurrence, while the remaining patients showed no signs of disease recurrence.

## 5 Discussion

Owing to the superiority of direct and straight corridors over retroinfundibular, prepontine and interpeduncular regions, the EEA technique has been prevalently employed to resect lesions around these regions (10, 15–19). Compared with the transcranial approach, the EEA approach is less invasive and much safer in that the anterior communicating artery complex, optic apparatus and oculomotor nerve are avoided (20–23). To ensure a sufficient surgical field and to observe lesions located in these complex anatomical areas, the natural anatomical barrier observed via endonasal access should be eliminated, from which the PG poses the main obstacle and can be preserved by PGT (6, 7, 24).

Extradural PGT, which was first proposed by Silva et al. (25), is associated with the lowest risk of PG dysfunction. All the preceding procedures were performed without opening either the periosteal or meningeal duraas, which are natural protectors of the sellar and parasellar neurovascular structures. However, this technique limits the effectiveness of PGT, avoids inadvertent tearing of the CS, and is generally adopted as an auxiliary manoeuvre following dorsectomy and posterior clinoidectomy (4, 10, 15, 17, 26). The interdural PGT technique, which is essentially a transcavernous approach between the medial and lateral walls of the CS (11, 16, 27), has proven more effective than the extradural PGT technique in terms of leveraging the natural corridor to access the PCPs, oculomotor triangle and lateral interpeduncular fossa and replacing the PG to resect selected retroinfundibular craniopharyngiomas, para-/retro-/suprasellar extensions of chordomas, petroclival meningiomas, and retroclival epidermoid tumours. However, addressing significant CS bleeding and meticulous preservation of the cavernous ICA is technically challenging because of the direct opening of the CS (12, 28, 29).

Intradural PGT, which was first developed by Kassam et al. (10). and is favoured by the authors of this report, has apparent advantages over the purely extradural approach in that the surgical corridor is wider, transposition is more extensive, the blood loss volume is lower and severe neurovascular complications occur less often (4, 6, 12). Moreover, the intradural approach is more effective than the interdural approach when tumours around the interpeduncular and prepontine cisterns are resected. Kassam et al. first proposed that direct gland manipulation and venous drainage disruption are two major defects that may increase the likelihood of pituitary dysfunction (10, 11, 30). However, this misgiving proved to be unlikely due to a high rate (87.5%, 7/8) of pituitary function preservation in his article about intradural PGT for retroinfundibular lesions resection. Although transient hypopituitarism occurred in 61% (14/23) and DI occurred in 35% (8/23) of the patients in our study, these complications were mainly occurred in craniopharyngiomas, which correlate closer to pituitary stalk and hypothalamus injury, but not direct gland manipulation. Furthermore, these complications were completely resolved or significantly improved by several weeks of replacement therapy. Accordingly, intradural PGT is a safe and optimal technique for retroinfundibular lesion resection (24, 31, 32).

Our study introduces a minimally invasive approach for the resection of these lesions. The first feature of this minimally invasive procedure is hemitransposition. After the anterior aspect of the PG is freed by opening the outer and inner dural layers, the PG is transposed, followed by transection of only one side of the PG ligaments, the IHA, the dorsum clival dura and the diaphragm sellae. This procedure avoided extra manipulation of the contralateral side of the PG and vascular system. Although bilateral PG manipulation and IHA sacrifice have been reported to not cause postoperative pituitary dysfunction in 75% of patients (31). Up to 25% of patients still experienced negative clinical consequences, which was prominently greater than that of patients who underwent hemitransposition (8, 24, 33). The second feature of this minimally invasive procedure is the reasonable combination of dorsectomy and posterior clinoidectomy with intradural PGT. Intradural PGT with single dorsectomy was adopted for retroinfundibular lesions with inferior invasion into the prepontine areas, which was sufficient to achieve satisfactory EOR in this region. The authors do not advocate posterior clinoidectomy as a routine procedure when dealing with retroinfundibular lesions. Only lesions with lateral extension or involving the interpeduncular cistern were needed to remove the ipsilateral PCP. Although it was verified as implementable in our study, PCP resection was reported to have a 20% possibility of neurovascular structure damage in other studies (17).

Intradural PGT is associated with a low risk of postoperative endocrinological dysfunction. The incidences of permanent hypopituitarism and DI in this series were 35% (8/23) and 17% (4/23), respectively. We hypothesize that postoperative pituitary function is more likely related to pathological features and the extent of tumour resection. The rate of pituitary preservation was 86% (6/7) for noncraniopharyngioma lesions and 56% (9/16) during surgery for craniopharyngiomas. The extent of tumour resection was confirmed to be the most significant risk factor for pituitary dysfunction. Gland function was well preserved in all patients with residual tumours (mainly those with germ cell tumours and gliomas), whereas pituitary function was not well preserved, especially in those with completely resected giant craniopharyngiomas with severe hypothalamus involvement. These results are similar to those of Kassam’s study (10). No postoperative CSF leakage was observed in this series, and the incidence of this complication was lower than that reported in previous studies with similar extents of dura resection or third ventricle opening (23, 34–36). In addition to the standard multilayered reconstruction technique, we recommended 7 days of perioperative LD in cases of high-flow CSF leakage, which was confirmed to be an effective method for further reducing postoperative CSF leakage and meningitis rates (37–39).

## 6 Conclusions

In conclusion, patients with retroinfundibular lesions can be treated safely and effectively via intradural pituitary transposition combined with reasonable dorsectomy and posterior clinoidectomy. The combined approach is associated with satisfactory resection and low incidences of endocrine dysfunction and visual impairment. This technique can be applied only by neurosurgeons with significant experience.

## Data Availability

The original contributions presented in the study are included in the article/supplementary material. Further inquiries can be directed to the corresponding author.
